# Toward Metallized Pellets for Steelmaking by Hydrogen Cooling Reduction: Effect of Gas Flow Rate

**DOI:** 10.3390/ma17163896

**Published:** 2024-08-06

**Authors:** Wanlong Fan, Zhiwei Peng, Ran Tian, Guanwen Luo, Lingyun Yi, Mingjun Rao

**Affiliations:** School of Minerals Processing and Bioengineering, Central South University, Changsha 410083, China; 225611071@csu.edu.cn (W.F.); 215601041@csu.edu.cn (R.T.); 225601020@csu.edu.cn (G.L.); yilingyun@csu.edu.cn (L.Y.); mj.rao@csu.edu.cn (M.R.)

**Keywords:** hydrogen cooling reduction, hydrogen flow rate, metalized pellets, porosity

## Abstract

This study proposed a strategy to prepare metalized pellets for direct steelmaking by hydrogen cooling reduction (HCR) of iron ore pellets with a focus on the effect of H_2_ flow rate on the process. It was demonstrated that increasing H_2_ flow rate could effectively enhance the reduction performance of iron ore pellets. However, due to the influence of the countercurrent diffusion resistance of gas molecules, too high H_2_ flow rate no longer promoted the reduction of the pellets when the maximum reduction rate was reached. The reduction swelling index (RSI) of the pellets initially increased and then decreased with increasing H_2_ flow rate. This change was associated with the decreased content of Fe_2_SiO_4_ in the metalized pellets and the changes in porosity and iron particle size. The compressive strength (CS) decreased continuously, showing a sharp decline when the H_2_ flow rate reached 0.6 L/min. It was attributed to the significant increases in porosity and average pore size of the metalized pellets, with the presence of surface cracks. When the H_2_ flow rate was 0.8 L/min, the metalized pellets had the optimal performance, namely, reduction degree of 91.45%, metallization degree of 84.07%, total iron content of 80.67 wt%, RSI of 4.66%, and CS of 1265 N/p. The findings demonstrated the importance of controlling the H_2_ flow rate in the preparation of metallized pellets by HCR.

## 1. Introduction

The iron and steel industry is an energy-intensive sector with substantial resource consumption [1]. It uses coal or coke as an energy resource which leads to significant CO_2_ emission [2,3]. This is particularly serious in the long process of steel production, namely the blast furnace-basic oxygen furnace (BF-BOF) process [4,5]. For cleaner and more efficient production, the short process, namely the direct reduction-electric arc furnace (DR-EAF) process, has been receiving increasing attention. It offers the advantages of higher efficiency and better sustainability, aligning with the future development of the iron and steel industry [6].

The DR-EAF process necessitates the reduction of iron ore pellets, which are produced from iron concentrates after pelletizing, drying, preheating (500–1100 °C), and roasting (1150–1400 °C) successively to obtain direct reduced iron (DRI) or metalized pellets for EAF smelting to produce crude steel [7,8]. It relies on reducing agents such as coal, natural gas, and H_2_. Among these, H_2_ stands out as an exceptionally clean reducing agent with superior reduction capability [9]. In recent years, with the maturation of “green hydrogen” technologies from clean energy, such as wind and water, the industrial application of using H_2_ for iron ore reduction to produce DRI has gradually become feasible [10,11]. Consequently, fostering the development and application of hydrogen-based direct reduction processes is vital for achieving “dual carbon” goals and producing high-quality steel.

For hydrogen-based direct reduction technologies, many studies have been reported [10,12,13]. These studies primarily focused on the effects of factors such as reduction temperature, reduction time, gas concentration, and pellet porosity on the product properties [14,15,16,17]. It was reported that elevating temperature could significantly enhance the reduction rate. However, excessively high temperature could result in an increase in pellet swelling, consequently reducing pellet strength [18]. For reduction time, its selection was of paramount importance for obtaining qualified metalized pellets with high production efficiency. For reducing gas like H_2_, its concentration was also found to be important because low H_2_ concentration would limit the reduction rate of the pellets, while excessively high concentration might induce pellet cracking [16]. For pellet porosity, increasing it properly would enhance contact areas between reducing gas molecules and iron oxides, thereby improving reduction efficiency [17]. These reports demonstrated that many factors controlled the hydrogen reduction process. However, there are rare reports on the influence of gas flow rate on the reduction performance of iron ore pellets.

Recently, the authors’ group proposed a method to produce metalized pellets by reduction of hot iron ore pellets after roasting (called roasted or oxidized pellets with temperatures higher than 1000 °C) during their cooling process in H_2_ [19]. This method was named hydrogen cooling reduction (HCR) and was expected to utilize the heat of the roasted pellets for reduction to enhance energy utilization efficiency. By following this concept, this study aimed to assess the influence of H_2_ flow rate on the reduction performance of iron ore pellets by examining the variations of the reduction degree, metallization degree, reduction swelling index (RSI), compressive strength (CS), phase composition, and microstructure of the pellets.

## 2. Materials and Methods

### 2.1. Raw Materials

**Figure 1:**
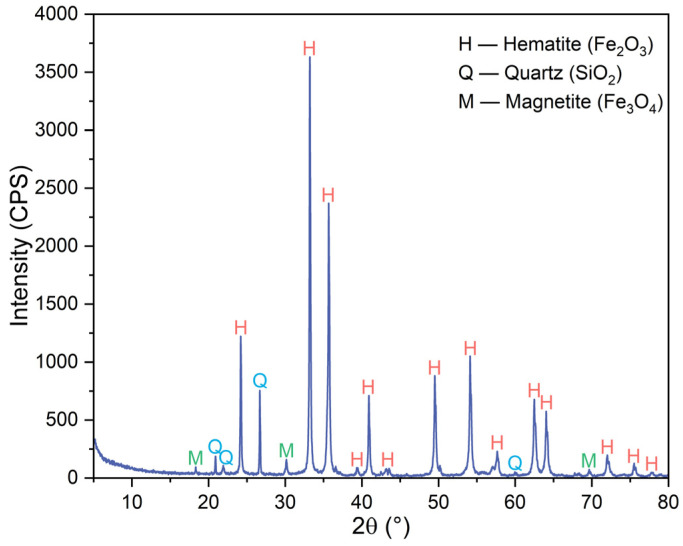
XRD pattern of iron ore pellets.

The main raw material used in this study was iron ore pellets. The pellets were prepared by drying of green pellets produced via traditional pelletizing of magnetite concentrate with the addition of 1.5% bentonite as the binder at 105 °C for 12 h, followed by preheating at 900 °C for 10 min, and then roasting at 1150 °C for 10 min. They had the bulk density of 3.31 g/cm^3^, porosity of 26.87%, compressive strength of 2100 N/p, and diameter of 14–16 mm. Table 1 shows their main chemical composition. They had total iron content of 64.00 wt%, SiO_2_ content of 8.51 wt%, and FeO content of 1.48 wt%. As shown by their XRD pattern in Figure 1, they were constituted by mainly hematite (Fe_2_O_3_), with small amounts of quartz (SiO_2_) and magnetite (Fe_3_O_4_).

### 2.2. Experimental Methods

#### 2.2.1. Direct Reduction

**Figure 2:**
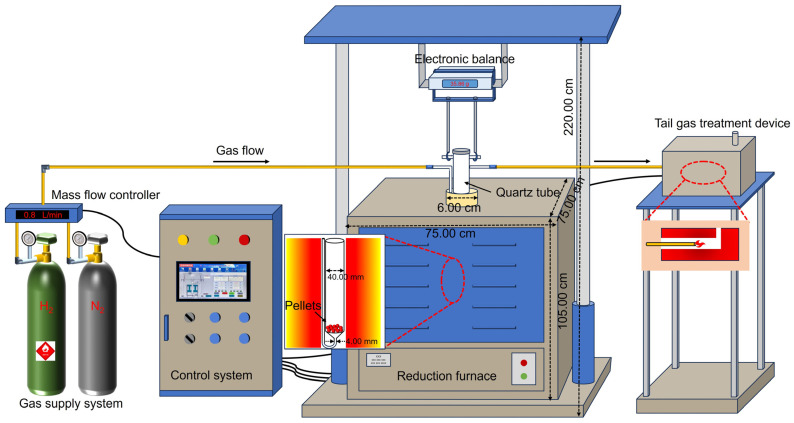
Schematic illustration of the experimental setup for reduction of iron ore pellets.

For each test, approximately 40 g of iron ore pellets were loaded in a vertical tube furnace (Figure 2) for reduction. Initially, the furnace temperature was elevated from room temperature to 1150 °C with the ramp rate of 10 °C/min. In this stage, N_2,_ with the flow rate of 1.0 L/min was introduced as the protective gas. After the pellets were kept at this temperature for 30 min, the gas was switched to H_2_ for HCR with the cooling rate of 3.7 °C/min, which was maintained using supplementary heat provided by the furnace until the temperature of the pellets decreased to 450 °C. These parameters were selected based on the preliminary experiments. During this process, different flow rates of H_2_, namely 0.2 L/min (case 1), 0.4 L/min (case 2), 0.6 L/min (case 3), 0.8 L/min (case 4), and 1.0 L/min (case 5), were selected to evaluate the effect of H_2_ flow rate on the reduction performance of the pellets. After the pellet temperature reached 450 °C, the gas was switched back to N_2_ with the flow rate of 1.0 L/min. The pellets after reduction, i.e., metalized pellets, were cooled naturally to room temperature for various characterizations. Note that throughout the experimental process, all gas flow rates were controlled accurately using a mass flow controller.

#### 2.2.2. Characterizations

The reduction degree and metallization degree of the pellets were determined according to the Chinese National Standard Test Method GB/T 24236–2009 [20]. The total iron content and CS were measured according to the Chinese National Standard Test Methods GB/T 6730.5–2022 [21] and GB/T 14201–2018 [22], respectively. The RSI values of the pellets were measured using the drainage method with anhydrous ethanol as the medium [23]. The phase compositions of the pellets were determined using an X-ray diffractometer (XRD; D8 Advance, Bruker, Hanau, Germany) and analyzed using the software Jade 9.0 (Materials Data Inc., Livermore, CA, USA). The contents of different phases of the metalized pellets were determined by Rietveld full-spectrum fitting based on the XRD patterns [24]. The microstructure changes of the pellets, which preliminarily underwent cold mounting in resin and polishing, were determined using a scanning electron microscope (SEM; Sigma HD, Zeiss, Göttingen, Germany) equipped with an energy dispersive X-ray detector (EDS; EDAX Inc., Mahwah, NJ, USA). The size and distribution of metallic iron particles in the pellets were measured using the software Image J 2.0 (National Institutes of Health, Bethesda, MD, USA). The pore size distribution, average pore size, and porosity of the pellets were measured using a mercury intrusion porosimeter (Autopore IV 9500, Micromeritics, Norcross, GA, USA). The porosities of various areas of the pellets were determined by Image J 2.0, in which 10 independent SEM images were selected for each region, and the mean values were used based on the measurements.

## 3. Results and Discussion

### 3.1. Reduction Behavior

**Figure 3:**
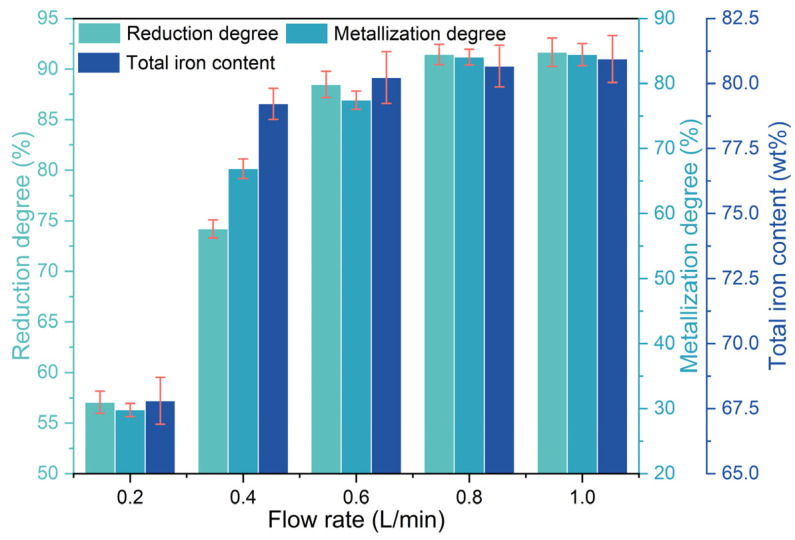
Effect of H_2_ flow rate on the reduction degree, metallization degree, and total iron content of the metalized pellets.

The reduction degree, metallization degree, and total iron content of the metalized pellets obtained by reduction with different H_2_ flow rates are shown in Figure 3. As the H_2_ flow rate increased from 0.2 L/min (case 1) to 0.8 L/min (case 4), the reduction degree, metallization degree, and total iron content of the metalized pellets increased from 57.05%, 29.81%, and 67.80 wt% to 91.45%, 84.07%, and 80.67 wt%, respectively, indicating increments of 34.40%, 54.26%, and 12.87 wt%. By increasing the H_2_ flow rate to 1.0 L/min, i.e., case 5, the reduction degree, metallization rate, and total iron content of the metalized pellets increased by only 0.21%, 0.39%, and 0.27 wt%, respectively. Due to the influence of the H_2_ flow rate, the reduction process was governed by both gas diffusion and chemical reaction [25,26]. Increasing the H_2_ flow rate enhanced both the diffusion rate of water vapor from the pellets and the contact areas between H_2_ molecules and the pellets, thereby promoting reduction of the pellets. However, the countercurrent diffusion resistance of H_2_ and water vapor molecules in the pellet pores, as well as the number of reaction sites within the pellets, remain unaffected [25]. By further increasing the H_2_ flow rate, the water vapor concentration within the pellets gradually approached a constant value [25]. Therefore, when the H_2_ flow rate exceeded 0.8 L/min, i.e., case 5, there was no significant enhancement of reduction performance of the pellets.

**Figure 4:**
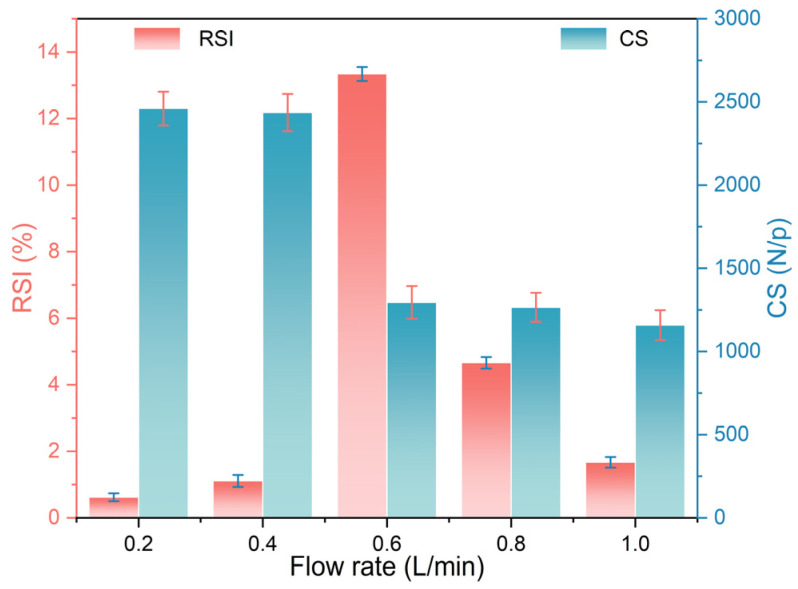
Effect of H_2_ flow rate on the RSI and CS of the metalized pellets.

Figure 4 shows the effect of H_2_ flow rate on the RSI and CS of the pellets. According to the results of cases 1–5, with increasing H_2_ flow rate, the RSI initially increased and then decreased. When the H_2_ flow rate increased from 0.2 L/min in case 1 to 0.6 L/min in case 3, the value of RSI increased from 0.62% to 13.34%. By further increasing the H_2_ flow rate to 1.0 L/min, i.e., case 5, the RSI decreased to 1.67%. During the initial reduction stage, the lattice transformation caused by the reduction of Fe_2_O_3_ to Fe_3_O_4_ resulted in an increase in pellet volume, accompanied by an increase in RSI [27,28]. As the H_2_ flow rate increased, the total amount of H_2_ involved in the reaction with iron ore pellets per unit time increased, leading to a higher reduction rate [16]. Therefore, the RSI increased initially and then decreased. As the H_2_ flow rate increased from 0.2 L/min (case 1) to 1.0 L/min (case 5), the value of CS of the metalized pellets decreased from 2460 N/p to 1158 N/p. In particular, when the H_2_ flow rate increased from 0.4 L/min (case 2) to 0.6 L/min (case 3), the CS decreased significantly by 1141 N/p, which correlated with a sharp increase in RSI [29]. The decreased CS of the metalized pellets was believed to be associated with relevant phase transformation and structural changes.

**Figure 5:**
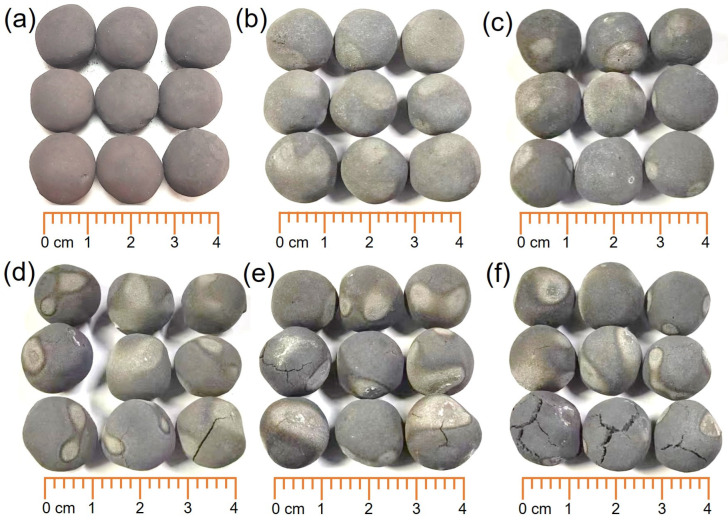
Macroscopic morphologies of (**a**) iron ore pellets and the metalized pellets obtained by reduction with different H_2_ flow rates: (**b**)—0.2 L/min, (**c**)—0.4 L/min, (**d**)—0.6 L/min, (**e**)—0.8 L/min, and (**f**)—1.0 L/min.

Figure 5 shows the macroscopic morphologies of iron ore pellets after reduction with different H_2_ flow rates. When the H_2_ flow rate was no more than 0.4 L/min, i.e., in cases 1 and 2, the resulting metalized pellets maintained intact structures and smooth surfaces. It indicated that with low H_2_ flow rates, there were no significant changes in the morphologies of the pellets. In cases 3–5, as the H_2_ flow rate further increased, more cracks appeared on the surfaces of the metalized pellets. The small H_2_ molecules enabled rapid penetration into the interior of the pellets, facilitating the reactions with iron oxides to form metallic iron layers and thus reducing the porosity of the pellets [16]. However, when the rate of water vapor generation exceeded its rate of outward diffusion, the internal gas pressure in the pellets increased, leading to pellet cracking. Therefore, for reduction with high H_2_ flow rates, the pellets were more prone to cracking, resulting in structural damage and, thus, lower strength.

### 3.2. Phase Transformation

**Figure 6:**
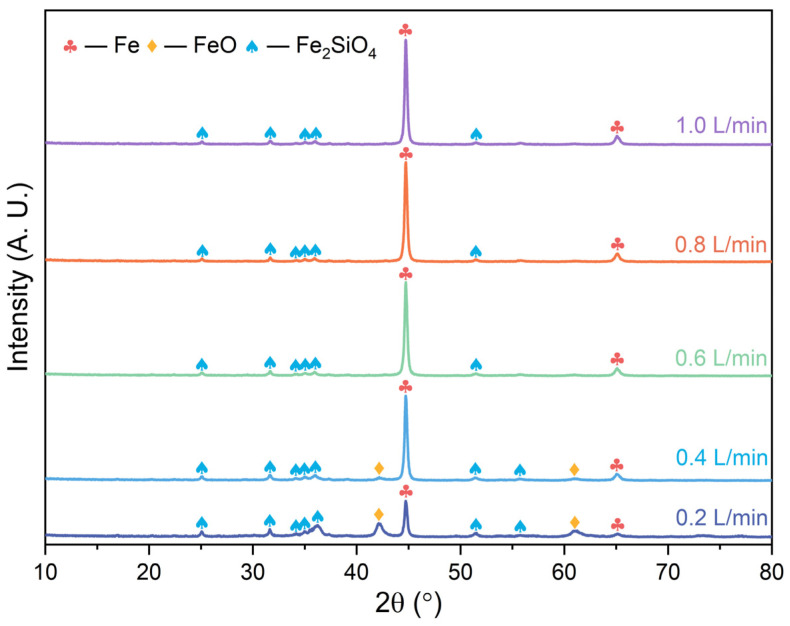
XRD patterns of the metalized pellets obtained by reduction with different H_2_ flow rates.

The phase compositions of the metalized pellets obtained by reduction with different H_2_ flow rates were determined by their XRD patterns in Figure 6. The main phases of the metallized pellets were metallic iron (Fe), wüstite (FeO), and fayalite (Fe_2_SiO_4_). In cases 1–5, with increasing H_2_ flow rate, the diffraction peak intensity of Fe increased continuously, while the diffraction peak intensity of FeO and Fe_2_SiO_4_ decreased. When the H_2_ flow rate reached 0.6 L/min, i.e., case 3, the diffraction peaks of FeO disappeared. It was demonstrated that increasing the H_2_ flow rate promoted the reduction process.

**Figure 7:**
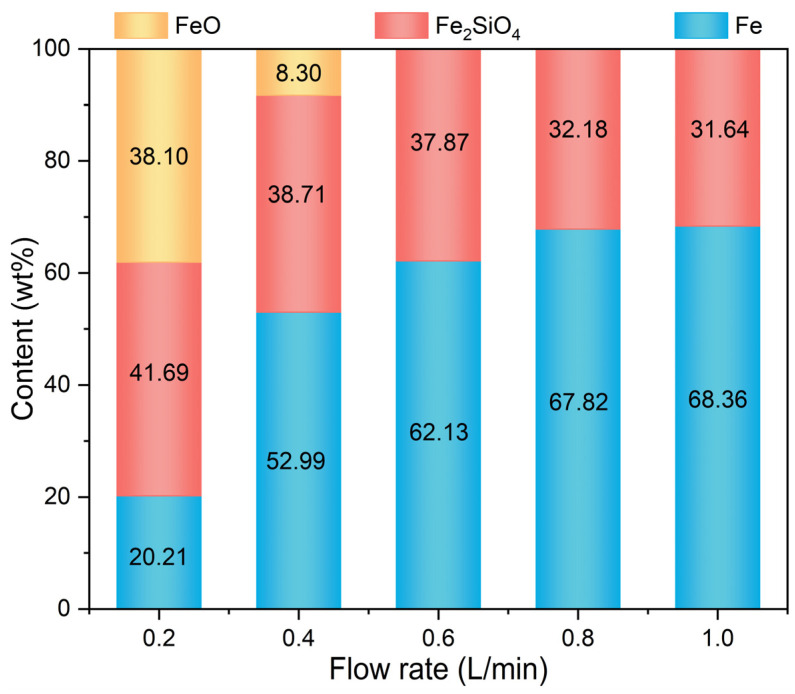
Contents of the main phases of the metalized pellets obtained by reduction with different H_2_ flow rates.

Figure 7 shows the variation of contents of different phases of the metalized pellets with H_2_ flow rate. As the H_2_ flow rate increased from 0.2 L/min (case 1) to 0.6 L/min (case 3), the content of Fe increased from 20.21 wt% to 62.13 wt%. Concurrently, the contents of FeO and Fe_2_SiO_4_ decreased from 38.10 wt% and 41.69 wt% to 0 wt% and 37.87 wt%, respectively. When the H_2_ flow rate further increased to 1.0 L/min, i.e., case 5, the content of Fe increased to 68.36 wt%, while that of Fe_2_SiO_4_ decreased to 31.64 wt%. This was because, with low H_2_ flow rates, such as cases 1 and 2, there were limited reactions of iron oxides, and a part of FeO remained unreduced. With high H_2_ flow rates, such as cases 3–5, the reduction of iron oxides was accelerated, producing more Fe through the reaction between FeO and H_2_ and less Fe_2_SiO_4_ from the reaction between residual FeO and SiO_2_ [30]. Consequently, with increasing H_2_ flow rate, the contents of FeO and Fe_2_SiO_4_ decreased.

### 3.3. Microstructural Evolution

**Figure 8:**
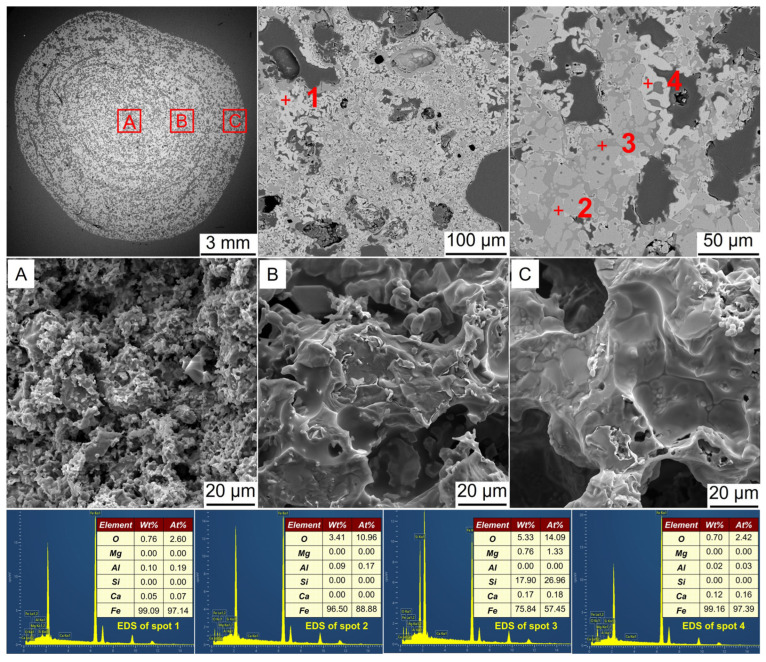
SEM-EDS analysis of the metalized pellets obtained by reduction with the H_2_ flow rate of 0.2 L/min: (**A**)–central area, (**B**)–1/4-diameter area, and (**C**)–edge area.

**Figure 9:**
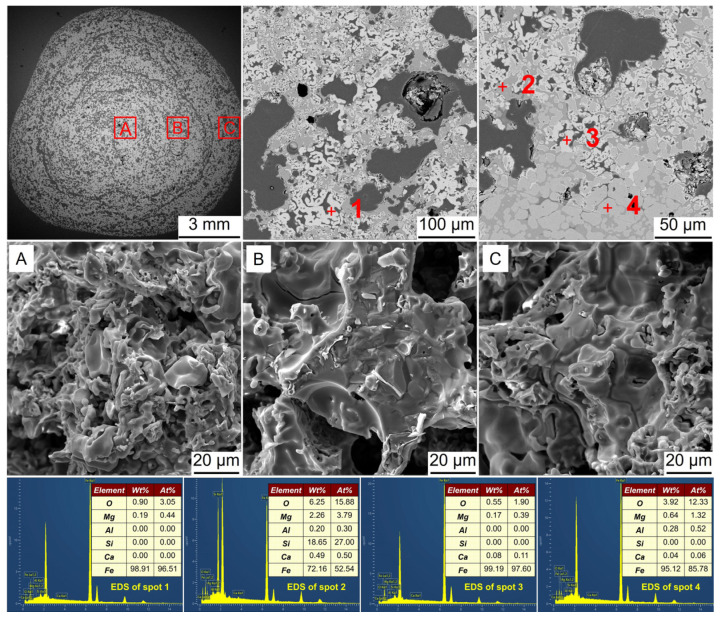
SEM-EDS analysis of the metalized pellets obtained by reduction with the H_2_ flow rate of 0.4 L/min: (**A**)–central area, (**B**)–1/4-diameter area, and (**C**)–edge area.

**Figure 10:**
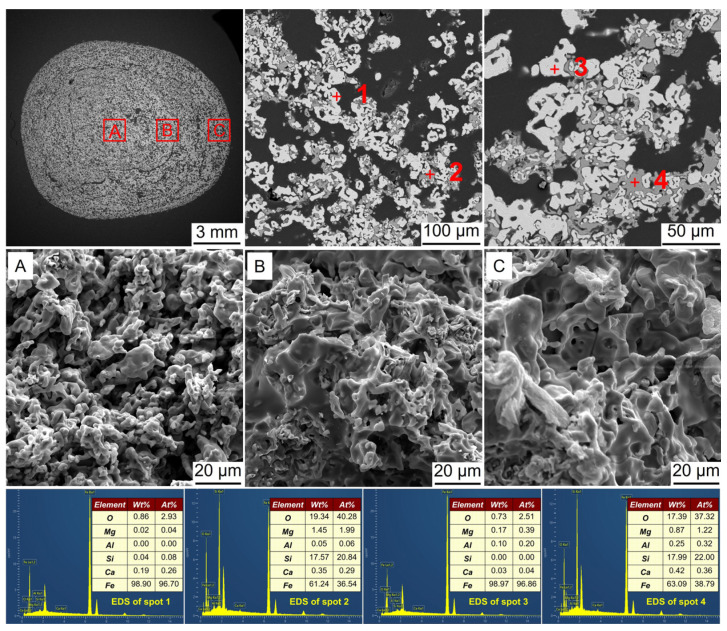
SEM-EDS analysis of the metalized pellets obtained by reduction with the H_2_ flow rate of 0.6 L/min in case 3: (**A**)–central area, (**B**)–1/4-diameter area, and (**C**)–edge area.

**Figure 11:**
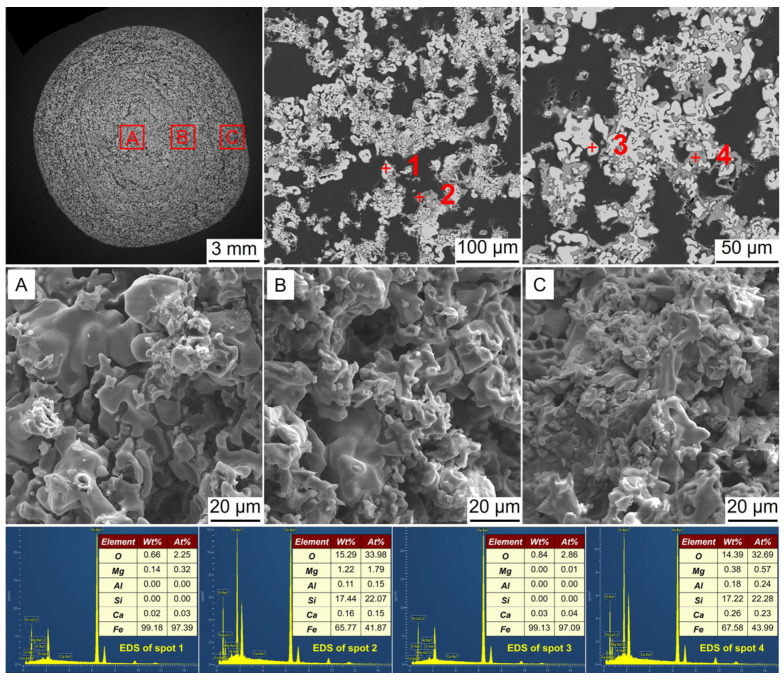
SEM-EDS analysis of the metalized pellets obtained by reduction with the H_2_ flow rate of 0.8 L/min: (**A**)–central area, (**B**)–1/4-diameter area, and (**C**)–edge area.

**Figure 12:**
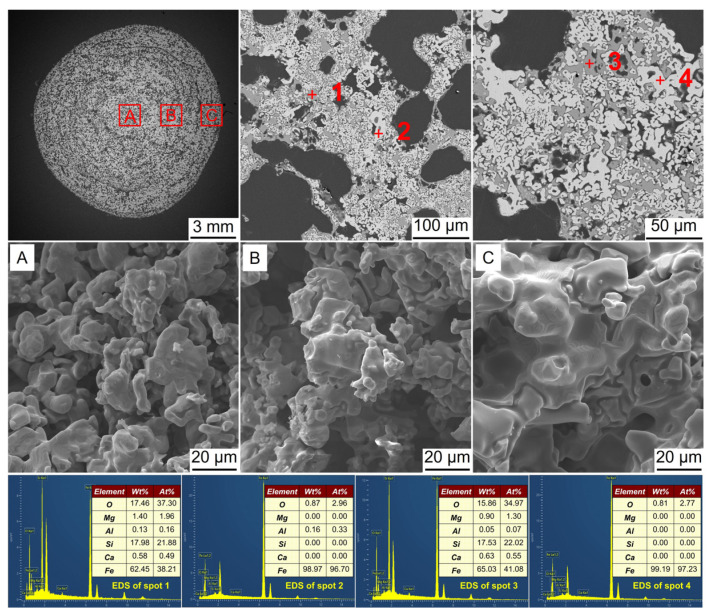
SEM-EDS analysis of the metalized pellets obtained by reduction with the H_2_ flow rate of 1.0 L/min: (**A**)–central area, (**B**)–1/4-diameter area, and (**C**)–edge area.

Along with the phase transformation, microstructural changes were expected to occur. Figure 8, Figure 9, Figure 10, Figure 11 and Figure 12 show the SEM-EDS analysis of metalized pellets obtained by reduction with different H_2_ flow rates. As shown in Figure 8, the compositions of spots 1 and 4 indicated that the predominant phase was Fe (metallic iron). At spot 2, the main elements were Fe and O, with a molar ratio of Fe to O close to 9, indicating the coexistence of both Fe and FeO in this area. At spot 3, the predominant elements were Fe, O, and Si, confirming the presence of Fe and Fe_2_SiO_4_. By observing the microscopic morphologies of the central, 1/4-diameter, and edge areas of the metalized pellets (denoted by A, B, and C, respectively), it was noted that the central area displayed a loose granular morphology while the edge area exhibited a relatively dense laminar structure with large pores. With a low H_2_ flow rate, such as 0.2 L/min, the inward diffusion of H_2_ into the pellet required a longer duration because it was impeded by the dense metallic iron layer formed in the outer layer after the reaction of H_2_ with iron oxides [31,32,33]. Consequently, the reduction degree in the pellet interior was relatively lower, leading to a sluggish growth of metallic iron particles. Additionally, it was observed that the metallic iron in the edge area existed in a relatively smooth form. This was attributed to the reduction by H_2_ in which metallic iron tended to precipitate in the form of a laminar structure rather than a whisker-like structure [34]. The laminar structure of metallic iron also contributed to a relatively low RSI of the metalized pellets, as reported before [35]. Furthermore, the formed dense metallic iron layer would help to increase CS of the pellets compared to the original pellets (2460 N/p vs. 2100 N/p).

Figure 9 shows the SEM-EDS analysis of the metalized pellets obtained by reduction with the H_2_ flow rate of 0.4 L/min. According to EDS analysis, at spots 1 and 3, Fe was identified, while at spot 2, Fe and Fe_2_SiO_4_ were observed. At spot 4, Fe and FeO were detected. Compared with the metalized pellets obtained at the H_2_ flow rate of 0.2 L/min, i.e., case 1, the metallic iron particles in the inner layer grew significantly. It suggested that increasing the flow rate facilitated the diffusion of H_2_ into the central area of the pellets, thereby achieving a better reduction performance. Additionally, a small number of metallic iron whiskers were also found in the central and 1/4-diameter areas, which partially accounted for the increase in RSI of the metalized pellets [12].

Figure 10 shows the SEM-EDS analysis of the metalized pellets obtained by reduction with the H_2_ flow rate of 0.6 L/min. According to the EDS analysis, at spots 1 and 3, Fe was observed, while at spots 2 and 4, both Fe and Fe_2_SiO_4_ were present. The absence of FeO indicated that FeO was fully reduced to Fe, which was consistent with the aforementioned XRD analysis. In the central area, there were a large number of metallic iron whiskers, leading to an increase in RSI of the metalized pellets. In the 1/4-diameter and edge areas, there were many small pores, probably resulting from the release of water vapor generated by the reduction of iron oxides inside the pellets. The internal structure of the pellets became highly porous, causing a considerable decrease in CS.

Figure 11 shows the SEM-EDS analysis of the metalized pellets obtained by reduction with the H_2_ flow rate of 0.8 L/min. At spots 1 and 3, Fe was observed, while at spots 2 and 4, Fe and Fe_2_SiO_4_ co-existed. In the central area, there existed only a small quantity of iron whiskers, contributing to the low RSI of the metalized pellets.

Figure 12 shows the SEM-EDS analysis of the metalized pellets obtained by reduction with the H_2_ flow rate of 1.0 L/min. Evidently, there were no significant structural changes in the metalized pellets. The metallic iron particles in the pellets continued to grow and no evident metallic iron whiskers were detected. It suggested that increasing the H_2_ flow rate could suppress the growth of metallic iron whiskers, reducing the RSI of the metalized pellets [32].

**Figure 13:**
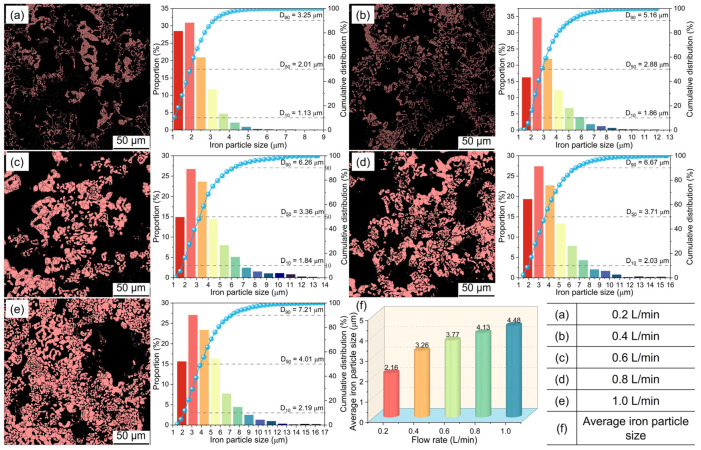
Distributions of metallic iron particles in the metalized pellets obtained by reduction with different H_2_ flow rates.

The aforementioned SEM analysis revealed notable size changes of metallic iron particles in the metalized pellets as the H_2_ flow rate changed. Figure 13 shows the size distributions of metallic iron particles. With increasing H_2_ flow rate from 0.2 L/min (case 1) to 1.0 L/min (case 5), the particle sizes corresponding to the cumulative distribution percentages reaching 10%, 50%, and 90% (D_10_, D_50_, and D_90_) increased from 1.13 μm, 2.01 μm and 3.25 μm to 2.19 μm, 4.01 μm, and 7.21 μm, respectively. The average metallic iron particle size of the metalized pellets increased from 2.16 μm to 4.48 μm, attributed to the faster reduction of iron oxides within the pellets when the H_2_ flow rate increased.

**Figure 14:**
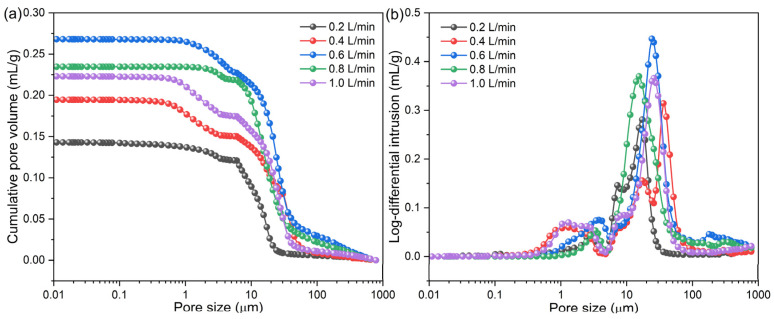
Pore size distributions of the metalized pellets obtained by reduction with different H_2_ flow rates: (**a**) cumulative pore volume and (**b**) log-differential intrusion.

**Figure 15:**
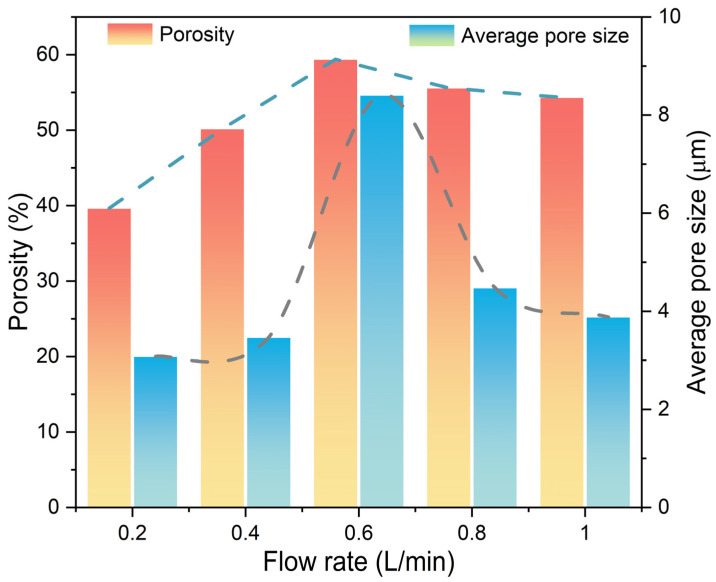
Effect of H_2_ flow rate on the porosity and average pore size of the metalized pellets.

The H_2_ flow rate also affected the pore structure in the metalized pellets. To clarify its effect, the variations of pore size distribution, porosity, and average pore size of the metalized pellets with H_2_ flow rate were measured. The results are shown in Figure 14 and Figure 15. As shown in Figure 14a, in cases 1–5, the cumulative pore volume in the metalized pellets initially increased and then decreased with increasing H_2_ flow rate. With the H_2_ flow rate of 0.6 L/min (case 3), the metalized pellets exhibited the largest cumulative pore volume. Figure 14b shows the log-differential intrusion curves of the metalized pellets. The pore size distribution in the metalized pellets primarily ranged from 5 μm to 60 μm. As shown in Figure 15, when the H_2_ flow rate increased from 0.2 L/min (case 1) to 0.6 L/min (case 3), the porosity and average pore size of the metalized pellets increased from 39.65% and 3.08 μm to 59.39% and 8.40 μm, respectively. When the H_2_ flow rate further increased to 1.0 L/min (case 5), the porosity and average pore size decreased to 54.33% and 3.88 μm, respectively. The variation of porosity and average pore size was basically consistent with the changing trend of RSI [12].

**Figure 16:**
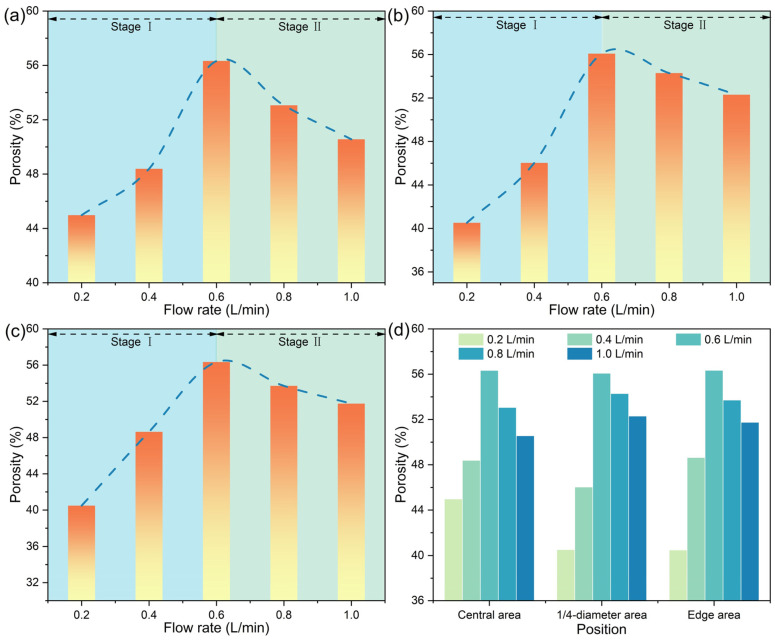
Variations of porosities of (**a**) central area, (**b**) 1/4-diameter area, and (**c**) edge area of the metalized pellets with H_2_ flow rate and (**d**) comparison of porosities between different areas.

With the progress of inward reduction of the pellets, there would be variations in the porosities of different positions of the pellets, as shown in Figure 16. According to the observed variation pattern in porosity within the metalized pellets, there were two distinct stages for all areas. In stage Ⅰ, i.e., in cases 1–3, the porosity increased with increasing H_2_ flow rate. Conversely, in stage Ⅱ, i.e., in cases 3–5, the porosity decreased. As shown in Figure 16d, when the H_2_ flow rate reached 0.6 L/min, i.e., case 3, the porosities of all areas of the metalized pellets were the highest, in agreement with the aforementioned SEM analysis.

Considering the reduction performance of the pellets, the proper H_2_ flow rate was found to be 0.8 L/min. Under this condition, the metalized pellets had the reduction degree of 91.45%, metallization degree of 84.07%, total iron content of 80.67 wt%, RSI of 4.66%, and CS of 1265 N/p. They were featured by high reduction and metallization degrees, low RSI, and modest CS.

Based on the above results, in cases 1–5, it was evident that increasing the H_2_ flow rate effectively enhanced the reduction efficiency of iron ore pellets. However, when the counterdiffusion resistance of water vapor reached a critical value, further increasing the H_2_ flow rate did not significantly enhance the reduction performance. In particular, under the condition of a high H_2_ flow rate, such as 1.0 L/min, the structure of metalized pellets became more susceptible to damage, producing evident cracks. Therefore, selecting an appropriate H_2_ flow rate is important for preparing high-quality metalized pellets with energy conservation.

## 4. Conclusions

In this study, the influence of H_2_ flow rate on the HCR behavior of iron ore pellets was investigated. The results indicated that increasing the H_2_ flow rate effectively enhanced the reduction efficiency of iron ore pellets. However, beyond a certain threshold, increasing the H_2_ flow rate did not significantly elevate pellet reduction performance because of the countercurrent diffusion resistance of water vapor molecules and limited reaction sites in the pellets. The RSI initially increased and then decreased with increasing H_2_ flow rate. This change was in association with the decrease in content of Fe_2_SiO_4_ in the metalized pellets and the changes in porosity and iron particle size. The CS decreased with increasing H_2_ flow rate, showing a sharp decline when the H_2_ flow rate reached 0.6 L/min, i.e., case 3. It was attributed to the significant increases in porosity and average pore size of the metallized pellets, with the formation of surface cracks. The content of Fe increased with increasing H_2_ flow rate, while the contents of FeO and Fe_2_SiO_4_ decreased accordingly. Meanwhile, the metallic iron particles in the metalized pellets gradually increased in size, which was one of the significant factors lowering the RSI of the metalized pellets. The porosity and average pore size of the metalized pellets initially increased and then decreased. The initial increases were associated with the evaporation of water vapor generated during the reduction process. After iron oxides were reduced to metallic iron, they began to grow in a lamellar form, which filled the internal pores of the pellets. Therefore, when the H_2_ flow rate increased to 0.6 L/min, both the porosity and average pore size decreased. When the H_2_ flow rate was 0.8 L/min, i.e., case 4, the optimal reduction performance could be achieved. The reduction degree, metallization degree, total iron content, RSI, and CS of the metalized pellets were 91.45%, 84.07%, 80.67 wt%, 4.66%, and 1265 N/p, respectively. Overall, this study elucidated the importance of controlling the H_2_ flow rate in the reduction of iron ore pellets during their cooling.

## Figures and Tables

**Table 1:** Main chemical composition of iron ore pellets (wt%).

TFe	SiO_2_	FeO	Al_2_O_3_	CaO	Na_2_O	MgO	K_2_O	P	S
64.00	8.51	1.48	0.42	0.18	0.18	0.15	0.10	0.045	0.0046

TFe–total iron content.

## Data Availability

The datasets presented in this article are not readily available because the data are part of an ongoing study. Requests to access the datasets should be directed to zwpeng@csu.edu.cn.
